# Targeting the Vim–PGI_2_ Pathway Enhances CD8^+^ T Cell‐Mediated Antitumor Immunity in Breast Cancer

**DOI:** 10.1155/humu/8880918

**Published:** 2026-04-28

**Authors:** Hong Quan, Lujing Shao, Qi Li, Chunyan Dong

**Affiliations:** ^1^ Shanghai East Clinical Medical College, Nanjing Medical University, Nanjing, Jiangsu, China, njmu.edu.cn; ^2^ Department of Breast surgery, Shanghai East Hospital, Tongji University School of Medicine, Tongji University, Shanghai, China, tongji.edu.cn; ^3^ Department of Oncology, Shanghai East Hospital, Tongji University School of Medicine, Tongji University, Shanghai, China, tongji.edu.cn

**Keywords:** arachidonic acid metabolism, breast cancer, CD8^+^ T cell activation, PGI_2_, Vim

## Abstract

Breast cancer is the most prevalent malignancy in women, and the limited effectiveness of current treatments highlights the need for novel immune regulatory mechanisms to improve long‐term survival. This study investigated the role of Vim in PGI_2_ synthesis and its impact on tumor immune regulation. Multiomics profiling revealed molecular alterations following Vim deletion, which were validated in murine breast cancer models using RT‐qPCR, Western blot, ELISA, and flow cytometry, with rescue experiments involving exogenous PGI_2_. The findings showed that Vim deletion downregulated arachidonic acid metabolism, reduced PTGIS expression, and significantly lowered PGI_2_ levels. Functional assays demonstrated that Vim deficiency enhanced T cell‐mediated antitumor immunity, evidenced by an increased proportion of CD8^+^ T cells, upregulation of cytotoxic genes (*Ifng*, *Gzmb*, *Tnf*, and *Klrd1*), and activation of inflammation‐related signaling pathways, as indicated by enhanced phosphorylation of ERK1/2 and p65. Both exogenous PGI_2_ supplementation and ozagrel treatment reversed these effects. In conclusion, the Vim–PGI_2_ axis is identified as a key regulator of CD8^+^ T cell immunity in breast cancer, representing a potential therapeutic target and a critical consideration in anticoagulant management during cancer immunotherapy.

## 1. Introduction

Breast cancer remains the most prevalent malignancy among women globally [1, 2]. Despite significant improvements in early detection leading to reduced mortality rates, chemotherapy′s therapeutic efficacy remains suboptimal, primarily due to interindividual variability in drug sensitivity, divergent treatment goals, and the nonspecific resistance of tumor cells [3–5]. These challenges emphasize the urgent need to identify novel immune regulatory mechanisms to enhance therapeutic outcomes and improve long‐term survival for breast cancer patients.

Emerging evidence suggests that metabolic pathways influence tumor biology by driving tumor cell proliferation while simultaneously reprogramming the tumor microenvironment (TME) to regulate immune activity, providing a critical entry point for understanding tumor immune evasion [6–9]. The arachidonic acid (AA) metabolic pathway is a key focus in inflammation and tumor biology. Its metabolites, in addition to contributing to vascular and platelet functions, play significant roles in immunosuppression and inflammation‐related tumorigenesis [10–14]. Prostacyclin (PGI_2_), a major downstream product of AA metabolism, has recently garnered attention. Previous studies have shown that PGI_2_ inhibits dendritic cell maturation, disrupts antigen presentation, promotes T cell exhaustion, and facilitates regulatory T cell (Treg) differentiation [15, 16]. Furthermore, a study revealed a significant association between the PGI_2_ synthase gene (PTGIS) and the infiltration of various immune cell subsets in colorectal cancer, suggesting that the PTGIR/PGI_2_ axis may contribute to the formation of the TME [17]. However, its precise role in tumor immunity remains incompletely understood.

Vimentin (Vim), a Class III intermediate filament protein, is critical for maintaining cytoskeletal integrity and facilitating the transition from epithelial to mesenchymal states [18–20]. Accumulating evidence indicates that aberrant Vim overexpression in tumors is closely linked to accelerated tumor growth, enhanced invasiveness, and poor clinical outcomes [21–24]. However, the precise mechanistic role of Vim in tumor progression is yet to be fully elucidated. Interestingly, recent studies suggest that Vim not only plays a role in immune cell adhesion and migration but is also involved in regulating lipid metabolism [25–28]. Given the central role of AA metabolism in lipid signaling and immune regulation, this study is aimed at investigating whether Vim governs AA metabolism and drives PGI_2_ production, and to explore how the Vim–AA–PGI_2_ axis orchestrates tumor immune responses, thereby offering new mechanistic insights into immune evasion and potential therapeutic targets.

## 2. Materials and Methods

### 2.1. Cell Culture

The EMT6 murine breast cancer cell line (RRID: CVCL_1923), derived from a mammary tumor in a BALB/c mouse, was used in this study. Both wild‐type (EMT6‐WT/Vim‐WT) and Vim‐knockout (EMT6‐KO/Vim‐KO) variants were obtained from the Stem Cell Bank of the Chinese Academy of Sciences (June 2023). Cells were cultured in DMEM supplemented with 10% fetal bovine serum and 1% penicillin–streptomycin at 37°C in a humidified 5% CO_2_ incubator. The identity of the cell line was authenticated via short tandem repeat profiling, and all cultures were confirmed to be mycoplasma‐negative prior to experimentation using the MycoAlert detection kit.

### 2.2. Tumor Xenograft Model

Cells in the exponential growth phase were harvested and resuspended in serum‐free DMEM at a density of 5–10 × 10^6^ cells/mL. Female C57BL/6 mice (6–8 weeks old) were sourced from the Shanghai Southern Model Animal Center and acclimatized for 1 week. All animal procedures were approved by the Animal Ethics Committee of Tongji University and conducted in compliance with institutional guidelines.

After disinfecting the dorsal skin with 75% ethanol, each mouse was subcutaneously injected with 0.1 mL of the cell suspension to establish a single tumor nodule. Mice were monitored daily. At predefined endpoints, animals were euthanized for blood and tumor collection. Tumor volume and body weight were recorded as indicated in the figure legends, with group sizes (*n*) specified therein.

### 2.3. Metabolomic Profiling by LC‐MS

Metabolite separation was performed using hydrophilic interaction chromatography with an ACQUITY UPLC BEH Amide column. The mobile phase consisted of (a) 25 mM ammonium acetate/ammonium hydroxide in water and (b) acetonitrile. The gradient elution conditions were as follows: 95% B for 0.5 min, followed by a linear decrease to 65% B over 4.5 min, a rapid drop to 40% B for 0.5 min, and re‐equilibration at 95% B for 2 min.

MS data were acquired in both positive and negative ESI modes. Source parameters included: Ion Source Gas 1 (50), Gas 2 (2), temperature (350°C), and IonSpray voltage (±3500/−2800 V). Full‐scan spectra (*m*/*z* 70–1200) were collected at a resolution of 60,000, followed by data‐dependent MS/MS acquisition with a 4 s dynamic exclusion window.

### 2.4. Metabolomic Data Analysis

Raw files were converted to mzXML format and processed using the XCMS package for peak detection, alignment, and annotation. Features detected in more than 50% of samples in at least one group were retained. Metabolite identification was based on matching accurate mass (< 10 ppm) and MS/MS spectra to an in‐house library of authentic standards.

Following sum normalization, multivariate analysis (PCA and OPLS‐DA) was performed using the ropls package in R. Model validity was assessed via cross‐validation and permutation testing. Metabolites with a VIP score > 1 and *p* < 0.05 (Student′s *t*‐test) were considered significantly altered. Enrichment analysis of KEGG pathways was performed using Fisher′s exact test.

### 2.5. Proteomic Analysis

Proteins were extracted, quantified, and subjected to in‐gel digestion using a filter‐aided sample preparation protocol. The resulting peptides were analyzed by LC‐MS/MS on a timsTOF Pro system coupled with an Evosep One LC. Peptides were separated on a reversed‐phase C18 column and analyzed using the PASEF acquisition method. Data were processed using MaxQuant (v1.6.14). Proteins exhibiting a fold change > 2 and *p* < 0.05 were classified as differentially expressed. Subcellular localization was predicted using the CELLO tool.

### 2.6. Transcriptomic Analysis

Total RNA was extracted, and library construction was performed using the NEBNext Ultra RNA Library Prep Kit. Paired‐end sequencing was conducted on an Illumina HiSeq platform. Raw reads were quality‐checked, aligned to the reference genome using STAR, and counted with HTSeq. Differential expression analysis was performed with DESeq2. Genes with |fold change| ≥ 2 and FDR < 0.05 were defined as differentially expressed genes (DEGs). Functional enrichment analysis of DEGs was conducted for GO terms and KEGG pathways [29–32].

### 2.7. Immune Cell Deconvolution

The relative abundances of 22 immune cell types within the TME were estimated from RNA‐seq data using the CIBERSORT algorithm with the LM22 signature matrix [33]. The LM22 leukocyte gene signature matrix was applied, and 1000 permutations were performed to ensure statistically robust estimates of immune cell fractions.

### 2.8. RT‐qPCR

Total RNA was reverse‐transcribed, and qPCR was performed using SYBR Green chemistry (R323‐01, Vazyme) on a Bio‐Rad CFX96 system. Relative expression was calculated using the 2^−*ΔΔ*Ct^ method with Gapdh as the internal control. All reactions were performed in triplicate across three independent experiments. The primer sequences are listed in Table S1.

### 2.9. Western Blot

Whole‐cell protein lysates were prepared using RIPA buffer (P0013B, Beyotime, China), and protein concentrations were determined with a BCA Protein Assay Kit (P0012, Beyotime). Proteins were separated by SDS‐PAGE and transferred to PVDF membranes. Membranes were probed with specific primary antibodies overnight, followed by incubation with horseradish peroxidase (HRP)‐conjugated secondary antibodies. Signals were detected by enhanced chemiluminescence and quantified using ImageJ software.

### 2.10. Flow Cytometry

For flow cytometric analysis, mouse peripheral blood mononuclear cells (PBMCs) were resuspended in staining buffer containing a predefined antibody cocktail and incubated at room temperature for 1 h. Following staining, cells were centrifuged at 1000 rpm for 5 min and resuspended in 250 *μ*L of ice‐cold PBS. Data acquisition was performed on a BD LSRFortessa flow cytometer (BD Biosciences, United States) using BD FACSDiva software, and data analysis was conducted using FlowJo (Tree Star, United States).

### 2.11. PGI_2_ Measurement

PGI_2_ levels were quantified using a commercial enzyme‐linked immunosorbent assay (ELISA) kit (D751007, Sangon Biotech, China) following the manufacturer′s instructions. In brief, standards and samples were added to microplate wells precoated with PGI_2_ antigen and incubated with a biotinylated anti‐PGI_2_ antibody. HRP‐conjugated streptavidin was subsequently added to form immune complexes. After extensive washing to remove unbound components, tetramethylbenzidine (TMB) substrate was introduced, producing a blue color that turned yellow upon the addition of the stop solution. Absorbance was measured at 450 nm using a microplate reader. PGI_2_ concentrations were determined from a standard curve, with values inversely correlated to the measured optical density. Each sample was analyzed in triplicate, and all experiments were independently repeated at least three times.

### 2.12. In Vivo Drug Administration

Tumor‐bearing mice were treated with either the PGI_2_ analog iloprost (administered via osmotic minipump at 0.3 mg/kg/min for 25 days) or the thromboxane synthase inhibitor ozagrel hydrochloride (administered by oral gavage at 10 mg/kg daily for 21 days). Control animals received the corresponding vehicle. Treatment regimens were based on established preclinical studies.

## 3. Results

### 3.1. Vim Deletion Alters AA Metabolism

**Figure 1:**
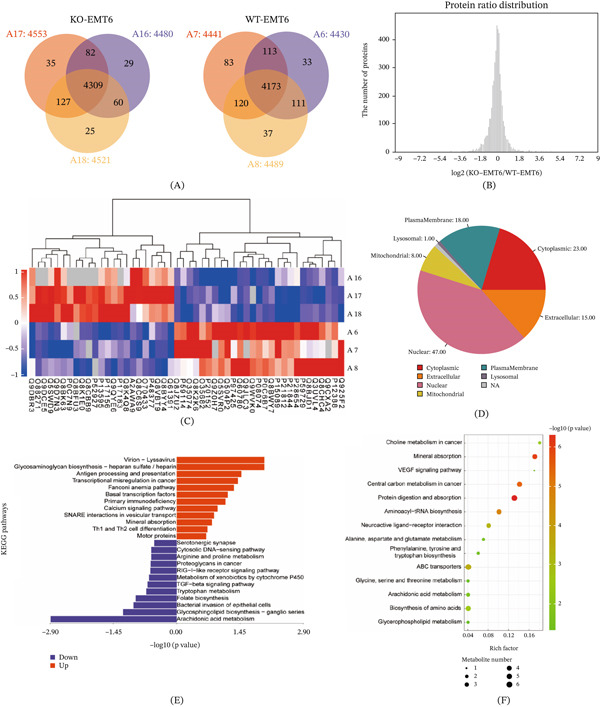
Differential proteomic and metabolomic enrichment analysis between Vim‐KO and Vim‐WT groups. (A) Venn diagram showing the number of proteins identified in the Vim‐KO and Vim‐WT groups. (B) Distribution of log_2_ fold change (FC) values in proteomic analysis. (C) Heatmap depicting differentially expressed proteins. (D) Subcellular localization distribution of differentially expressed proteins. (E) KEGG enrichment analysis of differentially expressed proteins. (F) KEGG enrichment analysis of differential metabolites. Proteomic (*n* = 3 biological replicates per group) and metabolomic (*n* = 5 per group) analyses were performed. Differential proteins and metabolites were identified based on log_2_(FC) and adjusted *p*‐values, as detailed in the Methods.

Bioinformatic processing of the mass spectrometry–based proteomic dataset was performed, and protein identification counts and quantified expression levels were summarized for each sample. The data revealed that most detected proteins overlapped within samples of the same group, suggesting minimal batch‐associated variation (Figure 1A). Comparative analysis between the Vim‐KO and Vim‐WT cohorts identified a set of proteins with significantly altered expression (Figure 1B,C). Subcellular localization profiling indicated that these differentially expressed proteins were predominantly localized to the nucleus (47%) and cytoplasm (23%), suggesting their involvement in processes such as DNA replication and transcription, as well as cellular metabolism and signal transduction (Figure 1D). KEGG pathway analysis further showed that these proteins were significantly enriched in antigen presentation and other immune‐associated pathways, whereas proteins related to AA metabolism were decreased (Figure 1E). Detailed results are provided in Table S1. Consistent with these findings, altered metabolites were also overrepresented in the AA pathway (Figure 1F).

Next, this study investigated how VIM regulates PTGIS expression. PTGIS is an endoplasmic reticulum (ER)‐resident membrane protein. Loss of Vim may disrupt ER structural continuity or signaling capacity, thereby disturbing calcium homeostasis and inducing mild ER stress. To alleviate the resulting cellular load, cells may reduce the production of specific proteins, including PTGIS. This interpretation is supported by GO enrichment analysis of upregulated genes (Figure S1a), which identified terms associated with ER stress responses and protein processing within the ER. These results suggest that Vim deficiency may affect AA biosynthesis through both direct and indirect mechanisms.

### 3.2. Reduced PGI_2_ Synthesis Upon Vim Deletion Enhances T Cell Immunity

**Figure 2:**
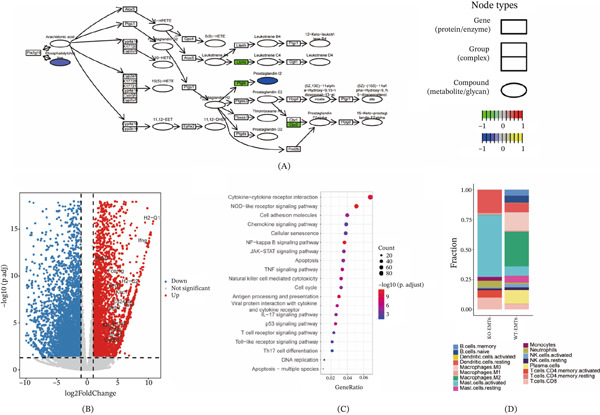
Reduced PGI_2_ synthesis upon Vim deletion enhances T cell immunity. (A) Arachidonic acid metabolism pathway. (B) Volcano plot of differentially expressed genes between Vim‐KO and Vim‐WT groups. (C) KEGG enrichment analysis of significantly upregulated genes in the Vim‐KO group compared with the Vim‐WT group. (D) Immune cell composition analysis using CIBERSORT. Transcriptomic analyses were conducted on *n* = 3 biological replicates per group. Statistical significance for differential gene expression was determined using appropriate models with multiple‐testing correction, as described in the Methods.

To explore the functional relationship between differentially expressed proteins and metabolites, both datasets were mapped onto KEGG pathways using the R package *pathview*. The analysis revealed that both differential proteins and metabolites were present in the AA metabolism pathway, with PTGIS (significantly downregulated in the Vim‐KO group) directly catalyzing the synthesis of PGI_2_ (also significantly downregulated in the Vim‐KO group) (Figure 2A). Additionally, the downstream impact of reduced PGI_2_ synthesis was examined using transcriptomic analysis. Compared with the Vim‐WT group, the Vim‐KO group exhibited significant upregulation of genes associated with T cell cytotoxicity and immunity, including *Ifng*, *Gzmb*, *Cd8a/b*, and *Klrd1* (Figure 2B). KEGG enrichment analysis also highlighted immune‐related pathways such as antigen presentation (Figure 2C). Conversely, among the downregulated genes, the most significantly enriched KEGG pathway was oxidative phosphorylation (FDR = 3.42e − 40), the primary energy‐producing pathway in cells. Additionally, the TCA cycle (FDR = 2.91e − 11) and glycolysis/gluconeogenesis (FDR = 0.002) pathways were also significantly downregulated (Figure S1b). This coordinated suppression of core metabolic pathways suggests that reduced PGI_2_ synthesis impairs cellular energy production. Notably, this metabolic constraint may create a permissive or compensatory environment that indirectly supports the observed upregulation of T cell cytotoxicity programs. Moreover, immune cell proportion estimation using the CIBERSORT deconvolution algorithm revealed that, compared with the Vim‐WT group, the Vim‐KO group exhibited increased fractions of CD8^+^ T cells, activated memory CD4^+^ T cells, and activated mast cells (Figure 2D). These results suggest that Vim deletion may alleviate immunosuppression and activate T cell–mediated immune responses by reducing PGI_2_ synthesis.

To further evaluate the clinical relevance of these findings, correlation analyses were performed using the TCGA breast cancer cohort (Figure S2). A significant positive correlation between VIM and PTGIS expression was observed, indicating that these two genes are coregulated in human tumors (Figure S2a). Survival analysis further demonstrated that patients with higher combined expression of VIM and PTGIS exhibited poorer overall survival compared with those with lower expression levels (Figure S2b), suggesting a potential protumorigenic role of this axis. Consistent with the experimental observations, PTGIS expression showed significant negative correlations with key cytotoxic immune markers, including IFNG, GZMB, and KLRD1 (Figures S2c, S2d, and S2e), implying that elevated PTGIS levels may be associated with suppressed cytotoxic immune activity. In addition, PTGIS expression was inversely correlated with the estimated infiltration of CD8^+^ T cells in the TME (Figure S2f). Collectively, these findings support the notion that the Vim–PTGIS–PGI_2_ axis contributes to an immunosuppressive TME in human breast cancer and validate the translational relevance of the mechanistic observations derived from the Vim‐KO model.

### 3.3. Vim Deletion Downregulates PTGIS Expression and Decreases PGI_2_ Levels

**Figure 3:**
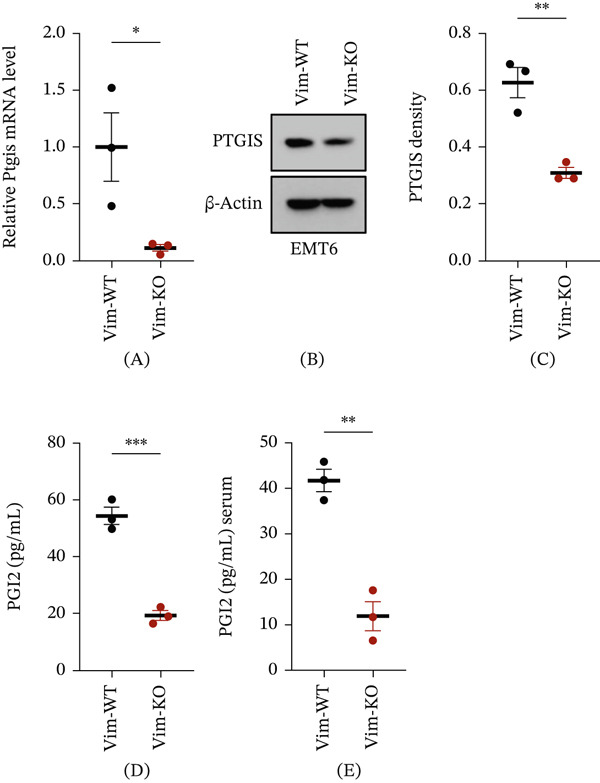
Vim deletion downregulates PTGIS expression and decreases PGI_2_ levels. (A) mRNA levels of *Ptgis* in EMT6 cell lines with Vim‐WT and Vim‐KO. (B–C) Immunoblotting and quantification of PTGIS in EMT6 cell lines with Vim‐WT and Vim‐KO. (D) PGI_2_ concentrations in the culture supernatants of Vim‐WT and Vim‐KO EMT6 cell lines under in vitro conditions. (E) PGI_2_ concentrations in the serum of C57BL/6 mice implanted with Vim‐WT or Vim‐KO EMT6 cells. *n* = 3 per group. Data are presented as mean ± SEM.  ^∗^ *p* < 0.05,  ^∗∗^ *p* < 0.01,  ^∗∗∗^ *p* < 0.001,  ^∗∗∗∗^ *p* < 0.0001. For two‐group comparisons, unpaired two‐tailed Student′s *t*‐tests were used.

To validate the omics‐derived findings, the impact of Vim loss on PTGIS expression and PGI_2_ production was assessed in the murine breast cancer cell line EMT6. Quantitative RT‐PCR revealed a significant reduction in PTGIS transcripts in Vim‐KO cells compared with Vim‐WT controls (Figure 3A). Consistent with the mRNA data, immunoblotting showed a marked decrease in PTGIS protein levels following Vim deletion (Figure 3B,C). As PTGIS (prostacyclin synthase) catalyzes PGI_2_ biosynthesis, this study further quantified PGI_2_ in conditioned media from Vim‐WT and Vim‐KO cultures, as well as in sera from C57BL/6 mice bearing tumors derived from the respective cell lines. PGI_2_ concentrations were significantly lower in the Vim‐KO group in both in vitro and in vivo settings compared with the Vim‐WT group (Figure 3D,E). These results corroborate the bioinformatic predictions, indicating that Vim deletion reduces PGI_2_ synthesis by suppressing PTGIS expression at both the transcript and protein levels.

### 3.4. Exogenous PGI_2_ Supplementation Reverses Vim Deletion–Induced Immune Activation

**Figure 4:**
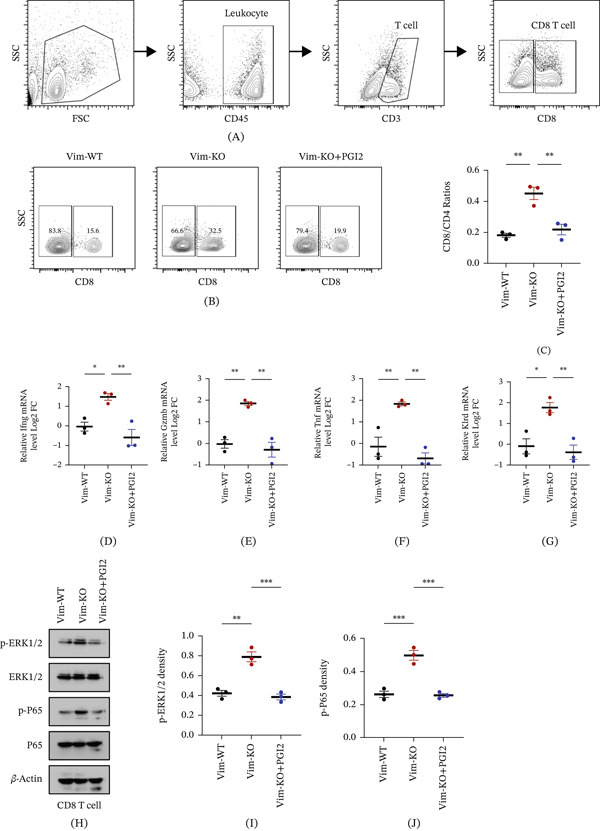
Exogenous PGI_2_ supplementation reverses Vim deletion–induced immune activation. (A) Flow cytometry‐gating strategy for CD4 and CD8 T cells in vivo. (B–C) CD8/CD4 ratios in C57BL/6 mice implanted with Vim‐WT or Vim‐KO EMT6 cells, or Vim‐KO cells with continuous PGI_2_ supplementation. mRNA levels of CD8 T cell cytotoxic markers *Ifng* (D), *Gzmb* (E), *Tnf* (F), and *Klrd1* (G) in the three groups. (H) Representative images and (i and j) quantitative analysis of downstream inflammatory signaling activation in CD8 T cells, including phosphorylation of ERK1/2 and P65. *n* = 3 per group. Data are presented as mean ± SEM.  ^∗^ *p* < 0.05,  ^∗∗^ *p* < 0.01,  ^∗∗∗^ *p* < 0.001,  ^∗∗∗∗^ *p* < 0.0001. For three‐group comparisons, one‐way ANOVA followed by Tukey′s post hoc test was applied unless otherwise indicated.

To explore the effect of Vim deletion on the tumor immune microenvironment, immune cell composition in C57BL/6 mice was examined. The CD8/CD4 ratio, a commonly used marker for assessing the intensity of antitumor immune responses, was significantly higher in mice implanted with Vim‐KO cells compared with those implanted with Vim‐WT cells (Figures 4A, 4B, and 4C), suggesting enhanced immune responses. Further isolation of CD8 T cells followed by RT‐qPCR demonstrated that Vim‐KO mice exhibited significantly elevated transcription of cytotoxic markers, including *Ifng*, *Gzmb*, *Tnf*, and *Klrd1*, relative to Vim‐WT controls (Figures 4D, 4E, 4F, and 4G). Western blot analysis also showed increased activation of inflammation‐related signaling pathways in CD8 T cells from Vim‐KO mice, as evidenced by enhanced phosphorylation of ERK1/2 and P65 compared with the Vim‐WT group (Figures 4H, 4I, and 4J). To determine whether this effect was mediated by reduced PGI_2_ secretion, the PGI_2_ analog iloprost was continuously administered via subcutaneous osmotic pumps. Notably, PGI_2_ supplementation reversed the immune activation phenotype in Vim‐KO mice, reducing the CD8/CD4 ratio, decreasing expression of cytotoxic markers, and diminishing activation of downstream inflammatory signaling pathways (Figures 4B, 4C, 4D, 4E, 4F, 4G, 4H, 4I, and 4J). These results demonstrate that Vim deletion enhances T cell–mediated immunity by reducing PGI_2_ secretion, whereas exogenous PGI_2_ supplementation can counteract this effect.

### 3.5. Ozagrel Suppresses Vim Deletion–Mediated Antitumor Immunity by Restoring PGI_2_ Signaling

**Figure 5:**
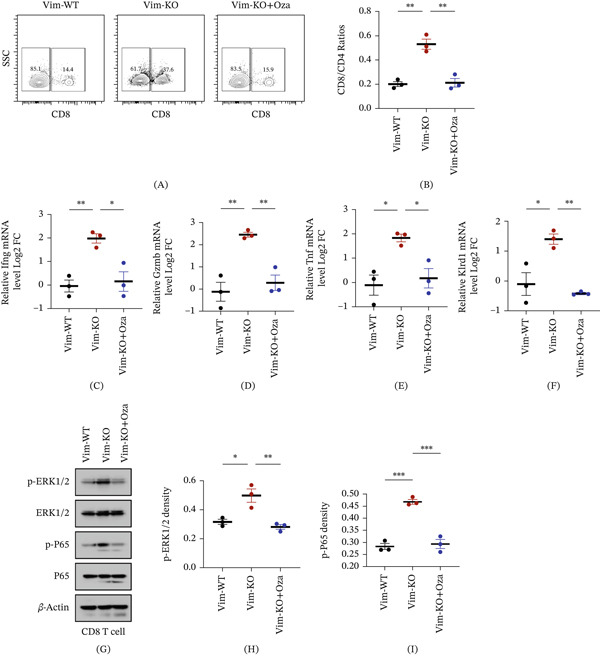
Ozagrel suppresses Vim deletion–mediated antitumor immunity by restoring PGI_2_ signaling. (A–B) CD8/CD4 ratios in C57BL/6 mice implanted with Vim‐WT or Vim‐KO EMT6 cells, or Vim‐KO cells with ozagrel treatment. Relative mRNA expression of CD8 T cell cytotoxic markers *Ifng* (C), *Gzmb* (D), *Tnf* (E), and *Klrd1* (F) in the three groups. (G) Immunoblot and (H and I) quantitative analysis of downstream inflammatory signaling in CD8 T cells, including phosphorylation of ERK1/2 and P65. *n* = 3 per group. Data are presented as mean ± SEM.  ^∗^ *p* < 0.05,  ^∗∗^ *p* < 0.01,  ^∗∗∗^p < 0.001,  ^∗∗∗∗^ *p* < 0.0001. For three‐group comparisons, one‐way ANOVA followed by Tukey′s post hoc test was applied.

Considering that cancer patients often experience a hypercoagulable state and require antithrombotic therapy, this study further explored how such drugs affect the immune responses mediated by Vim deletion. Ozagrel, an antithrombotic agent known to promote PGI_2_ release, was administered to mice implanted with Vim‐KO cells. The results showed that ozagrel treatment reduced the CD8/CD4 ratio, accompanied by significant downregulation of CD8 T cell cytotoxic marker genes, including *Ifng*, *Gzmb*, *Tnf*, and *Klrd1* (Figures 5A, 5B, 5C, 5D, 5E, and 5F). Consistent with this, Western blot analysis revealed decreased phosphorylation of ERK1/2 and P65, indicating suppression of inflammatory signaling pathway activation (Figures 5G, 5H, and 5I). These results demonstrate that ozagrel partially mitigates the immune activation induced by Vim deletion, highlighting the importance of carefully selecting antithrombotic agents for cancer patients to avoid compromising antitumor immunity.

## 4. Discussion

This study demonstrated that Vim sustains immunosuppression within the TME by regulating the PTGIS–PGI_2_ axis, whereas its deletion disrupts this pathway and enhances T cell–mediated antitumor immunity. Specifically, (i) Vim deletion downregulated the AA metabolic pathway, notably reducing PTGIS expression and subsequently decreasing PGI_2_ synthesis; (ii) reduced PGI_2_ levels were associated with enhanced T cell immunity, characterized by an increased proportion of CD8^+^ T cells, elevated expression of cytotoxic genes including *Ifng*, *Gzmb*, *Tnf*, and *Klrd1*, and enhanced phosphorylation of ERK1/2 and P65, indicating activation of effector functions; (iii) exogenous PGI_2_ supplementation or treatment with the antithrombotic agent ozagrel, which promotes PGI_2_ release and alters the prostanoid balance through thromboxane A2 (TXA2) synthase inhibition, reversed these effects and attenuated the antitumor immune responses induced by Vim deletion.

The metabolism of AA is increasingly recognized as a pivotal regulator of immune responses within tumors [34–37]. Its metabolites contribute to creating an immunosuppressive microenvironment through various mechanisms [38, 39]. Among these mediators, prostaglandin E2 (PGE2) is well established as a potent immunosuppressive agent that undermines antitumor immunity by impairing dendritic cell maturation, promoting Treg differentiation, and inducing T cell exhaustion [40–44]. In contrast, the immunomodulatory role of PGI_2_ remains less defined, as prior studies have mainly focused on its vasodilatory and antiplatelet effects [45, 46].

Cancer‐associated fibroblasts (CAFs) secrete significant amounts of PGI_2_, which suppress macrophage phagocytosis and, through interaction with the PGI_2_ receptor PTGIR, induces increased expression of genes in cancer cells that promote metastasis and angiogenesis [47]. However, the impact of PGI_2_ on antitumor T cell immunity has largely been unexplored. This study provides the first evidence that PGI_2_ directly inhibits the activation and functional capabilities of CD8^+^ T cells, thereby playing a negative regulatory role in the TME. Notably, whereas PGE2 exerts its immunosuppressive effects indirectly through antigen‐presenting cells and Tregs, our data suggest that PGI_2_ acts more directly on effector lymphocytes.

In contrast, the immunomodulatory properties of PGI_2_ are more complex and context‐dependent. Previous research has primarily focused on its vasodilatory and antiplatelet functions [45, 46], with both proinflammatory and anti‐inflammatory roles of PGI_2_ reported in nonmalignant settings. Recent studies have demonstrated that CAFs produce significant amounts of PGI_2_, which impairs macrophage phagocytosis and, via the PGI_2_ receptor PTGIR, induces prometastatic and proangiogenic gene expression programs in cancer cells [47]. Consistent with these findings, our study suggests that tumor‐derived PGI_2_ contributes to the suppressive nature of the tumor immune microenvironment. In the Vim‐KO setting, reduced PGI_2_ production was associated with an increased proportion of CD8^+^ T cells and enhanced expression of cytotoxic genes. However, restoration of PGI_2_ signaling through iloprost administration or ozagrel treatment reversed these immune responses. These data support a model in which PGI_2_–PTGIR signaling restrains CD8^+^ T cell‐mediated antitumor immunity.

This study also revealed that downregulation of PTGIS impaired cellular energy production. This metabolic constraint may create a permissive or compensatory microenvironment that indirectly supports the upregulation of T cell cytotoxicity programs. However, this remains a hypothesis, as direct experimental validation is still required. At present, it is unclear whether PGI_2_ acts directly on CD8^+^ T cells or indirectly through other immune or stromal cell populations. Elucidating this precise mechanism will be a critical focus of future research.

PGI_2_ primarily signals through the IP receptor (PTGIR), a Gs‐coupled G‐protein‐coupled receptor that activates cAMP/PKA signaling and can further engage downstream ERK1/2 and NF‐*κ*B (p65) pathways. The enhanced phosphorylation of ERK1/2 and P65 observed in our Vim‐WT and PGI_2_‐supplemented groups is consistent with canonical PTGIR signaling, providing a mechanistic link between the PTGIS–PGI_2_ axis and effector T cell function. Future studies using purified CD8^+^ T cells, PTGIR antagonists, and direct stimulation with PGI_2_ analogs will be necessary to dissect the relative contribution of direct PGI_2_ signaling to CD8^+^ T cell dysfunction in tumors.

These results suggest that the AA metabolic pathway does not simply support a uniform immunosuppressive program but rather establishes a complex regulatory framework through the coordinated actions of different metabolites, expanding the functional range of AA‐derived mediators. Our findings indicate that the AA metabolic pathway creates a dynamic regulatory network, in which distinct metabolites, including PGE2 and PGI_2_, may cooperate or counterbalance each other to fine‐tune antitumor immunity.

Vim is one of the most abundantly expressed and highly conserved members of the intermediate filament protein family [48, 49]. As a key component of the cytoskeleton, Vim forms intracellular filamentous networks that provide mechanical strength and structural stability to cells. Beyond its structural role, Vim has recently gained prominence as a traditional marker for epithelial–mesenchymal transition (EMT) [50]. EMT plays a critical role in embryonic development and tissue repair, and it is also a significant factor in tumor invasion, metastasis, and resistance to therapy [51, 52]. Furthermore, the widespread overexpression of Vim in various cancers and its close association with tumor progression and poor prognosis highlight its potential as a promising therapeutic target [21, 53]. This study reports for the first time that loss of Vim significantly reduces PGI_2_ production and enhances T cell–mediated antitumor immunity. This discovery establishes a direct link between Vim and the AA metabolic pathway, suggesting that interventions targeting this pathway may offer a novel strategy to overcome tumor immune resistance. As a cytoskeletal protein, Vim knockout may disrupt the physical connectivity or signaling of the ER, leading to disturbances in calcium homeostasis and triggering mild ER stress. To alleviate this stress, the synthesis of certain proteins, including PTGIS, may be downregulated. Follow‐up studies will aim to experimentally validate this hypothesis and clarify the specific mechanisms involved. Nevertheless, targeting the Vim–PTGIS–PGI_2_ axis may represent a promising strategy for overcoming tumor immune resistance.

Additionally, this study found that ozagrel, a widely used clinical antithrombotic drug, restored PGI_2_ levels, altered the prostanoid balance, and functionally reactivated PGI_2_‐dependent signaling, reversing the immune activation induced by Vim loss. This effect likely reflects a combined impact of reduced TXA2 and relatively enhanced PGI_2_ signaling, emphasizing the importance of the TXA2/PGI_2_ balance in shaping tumor immunity. These findings highlight the critical role of the Vim–AA–PGI_2_ axis in tumor immunology and its potential clinical relevance. For instance, PGI_2_–PTGIR signaling, along with downstream ERK1/2 and NF‐*κ*B activation, could intersect with established immunosuppressive pathways such as PD‐1/PD‐L1, potentially promoting the expression of exhaustion‐associated transcriptional programs or inhibitory receptors on T cells. Although PD‐1/PD‐L1 expression was not directly evaluated in this study, our data suggest that targeting the Vim–PTGIS–PGI_2_ axis may sensitize tumors to immune checkpoint blockade. For cancer patients, particularly those undergoing long‐term anticoagulant therapy, careful evaluation of the immunomodulatory effects of such drugs is essential to avoid compromising the efficacy of immunotherapy. In particular, the use of TXA2 synthase inhibitors like ozagrel in patients receiving immunotherapy should be considered in light of their potential to modulate prostanoid‐mediated immune responses within the TME.

This study has several limitations. The precise mechanisms by which Vim regulates PTGIS, including potential effects on transcription, translation, or protein stability, require further clarification. Additionally, AA metabolism generates multiple bioactive mediators, and Vim may modulate immune regulation through pathways beyond PGI_2_, warranting further investigation. First, the in vivo work was conducted exclusively using the EMT6 murine breast cancer model, which may not fully capture the biological diversity of human breast cancer or apply to other malignancies. The contribution of the Vim–PTGIS–PGI_2_ axis is likely context‐dependent, potentially varying by molecular subtype and TME. Therefore, replication in additional systems—such as human breast cancer cell lines, patient‐derived xenografts, and other murine models—is needed to establish the robustness and broader applicability of these findings. Second, although our data strongly suggest that tumor‐derived PGI_2_ suppresses CD8^+^ T cell–mediated antitumor immunity, the exact mechanisms by which Vim regulates PTGIS remain unclear. It is also uncertain whether PGI_2_ acts directly on CD8^+^ T cells or indirectly through other immune or stromal cell populations. Future studies using purified cell subsets and PTGIR modulation are necessary to address this question. Finally, the broad array of bioactive mediators produced by AA metabolism suggests that Vim may influence immune regulation through additional pathways beyond PGI_2_, which warrants further exploration.

## Author Contributions

Qi Li and Chunyan Dong conceived the project and participated in the study design and interpretation of the results. Hong Quan and Lujing Shao wrote the manuscript. Qi Li and Lujing Shao participated in the study design and helped draft the manuscript. Hong Quan and Chunyan Dong participated in data interpretation and provided a critical review of the manuscript. Hong Quan and Lujing Shao contributed equally to this work.

## Funding

This study was supported by Shanghai Leading Talent Program of Eastern Talent Plan (DFYCLJ02); Leading Talent Program by Shanghai Municipal Health Commission (2022LJ012); Shanghai 2023 “Science and Technology Innovation Action Plan” Outstanding Academic/Technical Leader Program (23XD1402800); Key Specialty Construction Project of Shanghai Pudong New Area Health Commission (PWZzk2022‐01); Shanghai Pudong New Area Traditional Chinese Medicine Inheritance Innovation Development Demonstration Pilot Project Construction (YC‐2023‐0403); Shanghai Pudong New Area Advanced Talent Cultivation Project of Integrated Traditional Chinese and Western Medicine (PDZY‐2024‐0701); New Quality Clinical Specialty Program of High‐end Medical Disciplinary Construction in Shanghai Pudong New Area (2025‐PWXZ‐01);Tongji University "Medicine + X" Interdisciplinary Research Project(2025‐0345‐ZD‐02).

## Disclosure

All authors reviewed and approved the manuscript.

## Conflicts of Interest

The authors declare no conflicts of interest.

## Supporting information


**Supporting Information** Additional supporting information can be found online in the Supporting Information section. Table S1: qPCR primer sequences. Figure S1: Functional enrichment analysis of differentially expressed transcripts in Vim‐KO versus Vim‐WT cells. (a) Dot plot of Gene Ontology (GO) enrichment analysis for upregulated genes. (b) Dot plot of KEGG pathway enrichment analysis for upregulated genes. Figure S2: Correlation of PTGIS expression with VIM, immune markers, CD8+ T cell infiltration, and patient survival in breast cancer (TCGA cohort). (a) Scatter plot showing a significant positive correlation between the mRNA expression levels of VIM and PTGIS (Spearman′s correlation test). (b) Kaplan–Meier survival curves comparing overall survival between patients with high versus low expression of VIM + PTGIS. Statistical significance was determined by the log‐rank test. (c–e) Scatter plots demonstrating the correlation between PTGIS expression and the expression of immune‐related genes: (c) IFNG (interferon‐gamma), (d) KLRD1 (a marker for natural killer cells), and (e) GZMB (Granzyme B). Spearman′s correlation test was applied. (f) Scatter plot showing a correlation between PTGIS expression and the estimated abundance of CD8+ T cell infiltration in the tumor microenvironment (Spearman′s correlation test).

## Data Availability

The data that support the findings of this study are available from the corresponding authors upon reasonable request.
