# Supplementary material for: Targeting the Vim–PGI2 Pathway Enhances CD8+ T Cell‐Mediated Antitumor Immunity in Breast Cancer
Source: Hum Mutat. 2026 Apr 28;2026:8880918. doi: 10.1155/humu/8880918 (PMC13122558; doi:10.1155/humu/8880918)
Supplement: Supplementary file 1 — Supporting Information Additional supporting information can be found online in the Supporting Information section. Table S1: qPCR primer sequences. Figure S1: Functional enrichment analysis of differentially expressed transcripts in Vim‐KO versus Vim‐WT cells. (a) Dot plot of Gene Ontology (GO) enrichment analysis for upregulated genes. (b) Dot plot of KEGG pathway enrichment analysis for upregulated genes. Figure S2: Correlation of PTGIS expression with VIM, immune markers, CD8+ T cell infiltration, and patient survival in breast cancer (TCGA cohort). (a) Scatter plot showing a significant positive correlation between the mRNA expression levels of VIM and PTGIS (Spearman′s correlation test). (b) Kaplan–Meier survival curves comparing overall survival between patients with high versus low expression of VIM + PTGIS. Statistical significance was determined by the log‐rank test. (c–e) Scatter plots demonstrating the correlation between PTGIS expression and the expression of immune‐related genes: (c) IFNG (interferon‐gamma), (d) KLRD1 (a marker for natural killer cells), and (e) GZMB (Granzyme B). Spearman′s correlation test was applied. (f) Scatter plot showing a correlation between PTGIS expression and the estimated abundance of CD8+ T cell infiltration in the tumor microenvironment (Spearman′s correlation test). [file HUMU-2026-8880918-s001.docx]

**
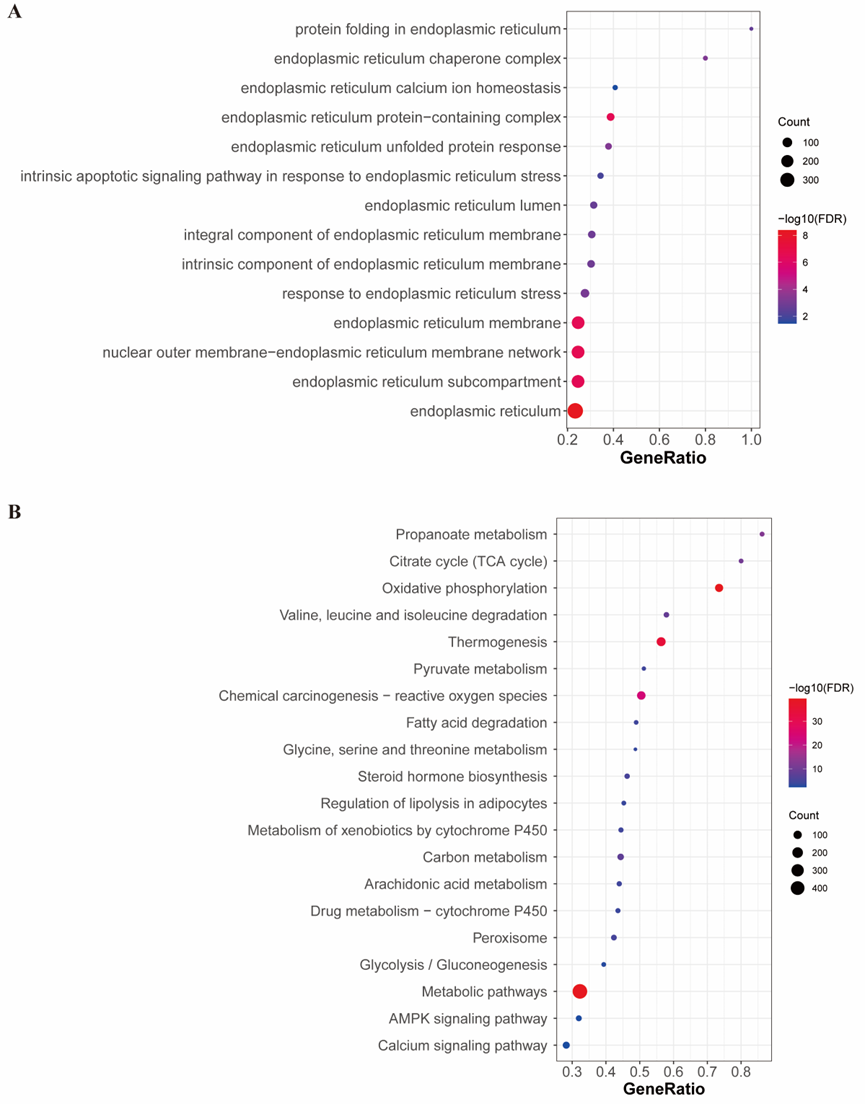
**

**Figure S1. Functional enrichment analysis of differentially expressed transcripts in Vim-KO versus Vim-WT cells.** (A) Dot plot of Gene Ontology (GO) enrichment analysis for upregulated genes. (B) Dot plot of KEGG pathway enrichment analysis for upregulated genes.


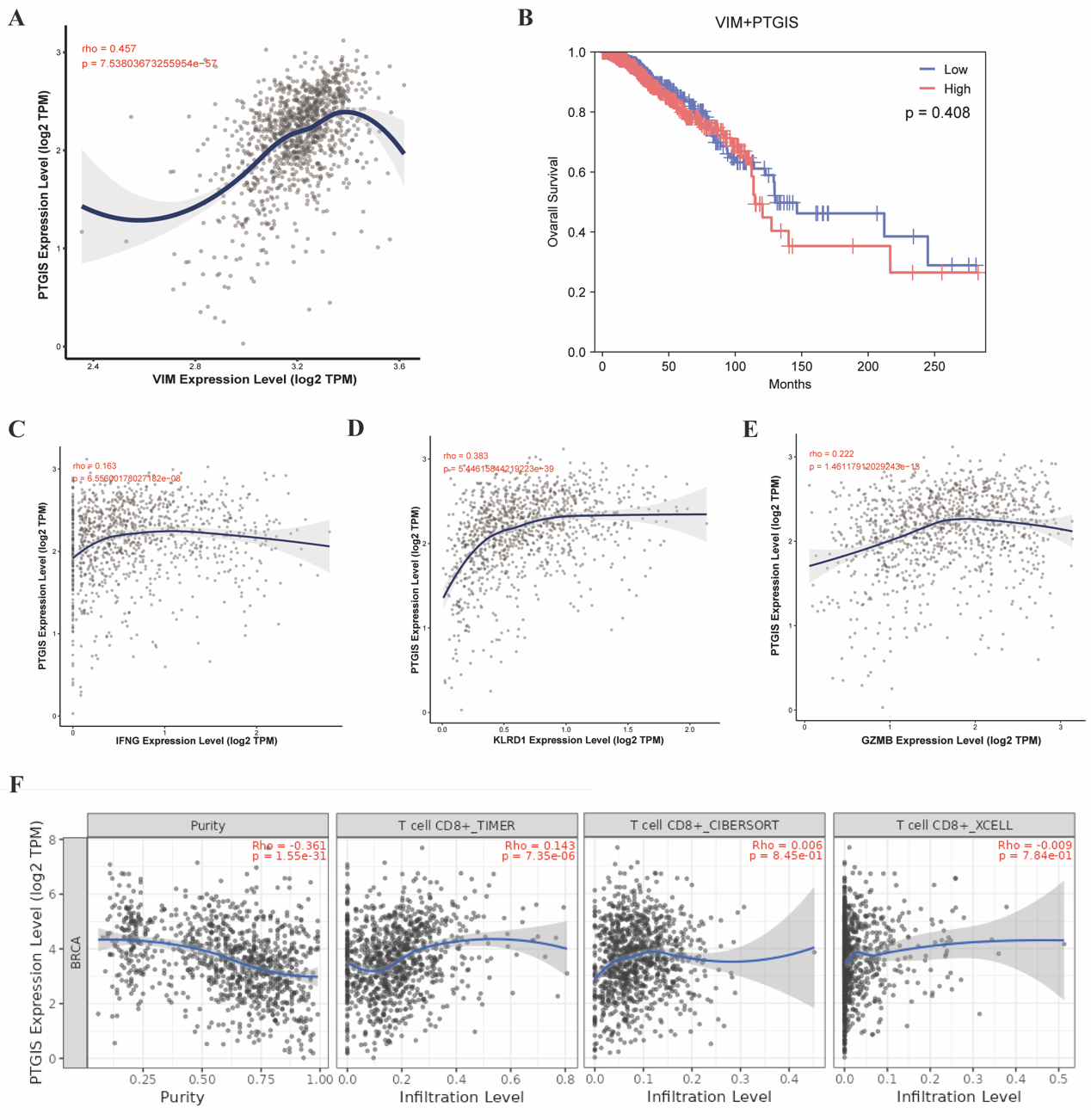


**Figure S2. Correlation of PTGIS expression with VIM, immune markers, CD8^+^ T cell infiltration, and patient survival in breast cancer (TCGA cohort).** (A) Scatter plot showing a significant positive correlation between the mRNA expression levels of VIM and PTGIS (Spearman’s correlation test). (B) Kaplan-Meier survival curves comparing overall survival between patients with high versus low expression of VIM+PTGIS. Statistical significance was determined by the log-rank test. (C-E) Scatter plots demonstrating the correlation between PTGIS expression and the expression of immune-related genes: (C) IFNG (Interferon-gamma), (D) KLRD1 (a marker for natural killer cells), and (E) GZMB (Granzyme B). Spearman’s correlation test was applied. (F) Scatter plot showing a correlation between PTGIS expression and the estimated abundance of CD8^+^ T cell infiltration in the tumor microenvironment (Spearman’s correlation test).

Table S1: qPCR primer sequences.

| Gene | Forward primer sequence (5’-3’) | Reverse primer sequence (5’-3’) |
| --- | --- | --- |
| *Ptgis* | GGAGACAGGTCTCCTTGAGTTC | AACATCCGCTGAGTGGACACGA |
| *Ifng* | CAGCAACAGCAAGGCGAAAAAGG | TTTCCGCTTCCTGAGGCTGGAT |
| *Gzmb* | CAGGAGAAGACCCAGCAAGTCA | CTCACAGCTCTAGTCCTCTTGG |
| *Tnf* | GGTGCCTATGTCTCAGCCTCTT | GCCATAGAACTGATGAGAGGGAG |
| *Klrd1* | GCAGAGATTTCTGTGCTTCGCAG | CATCCTCCCATAGCCAGGCATT |
| *Gapdh* | CATCACTGCCACCCAGAAGACTG | ATGCCAGTGAGCTTCCCGTTCAG |
